# Biotechnological enhancement of lactic acid conversion from pretreated palm kernel cake hydrolysate by *Actinobacillus succinogenes* 130Z

**DOI:** 10.1038/s41598-023-32964-z

**Published:** 2023-04-08

**Authors:** Nuraishah Abd Rahim, Abdullah Amru Indera Luthfi, Nurul Adela Bukhari, Jian Ping Tan, Peer Mohamed Abdul, Shareena Fairuz Abdul Manaf

**Affiliations:** 1 Department of Chemical and Process Engineering, Faculty of Engineering and Built Environment, Universiti Kebangsaan Malaysia, 43600 Bangi, Selangor Malaysia; 2 Research Centre for Sustainable Process Technology (CESPRO), Faculty of Engineering and Built Environment, Universiti Kebangsaan Malaysia, 43600 Bangi, Selangor Malaysia; 3 Energy and Environment Unit, Engineering & Processing Research Division, Malaysian Palm Oil Board (MPOB), 6, Persiaran Institusi, Bandar Baru Bangi, 43000 Kajang, Selangor Malaysia; 4 School of Energy and Chemical Engineering, Xiamen University Malaysia, Jalan Sunsuria, Bandar Sunsuria, 43900 Sepang, Selangor Darul Ehsan Malaysia; 5 School of Chemical Engineering, College of Engineering, Universiti Teknologi MARA, 40450 Shah Alam, Selangor Malaysia

**Keywords:** Biological techniques, Biotechnology

## Abstract

The aim of this study was to establish an improved pretreatment and fermentation method i.e. immobilized cells for high recovery of fermentable sugars from palm kernel cake (PKC) and its effects on fermentability performance by *Actinobacillus succinogenes* 130Z in the conversion of the fermentable sugar to lactic acid. The effects of oxalic acid concentrations (1–6% w/v) and residence times (1–5 h) on the sugar recovery were initially investigated and it was found that the highest mannose concentration was 25.1 g/L at the optimum hydrolysis conditions of 4 h and 3% (w/v) oxalic acid. The subsequent enzymatic saccharification of the pretreated PKC afforded the highest enzymatic digestibility with the recovered sugars amounting to 25.18 g/L and 9.14 g/L of mannose and glucose, respectively. Subsequently, the fermentability performance of PKC hydrolysate was evaluated and compared in terms of cultivation phases (i.e. mono and dual-phases), carbonate loadings (i.e. magnesium and sodium carbonates), and types of sugars (i.e. glucose and mannose). The highest titer of 19.4 g/L lactic acid was obtained from the fermentation involving *A. succinogenes* 130Z in dual-phase cultivation supplemented with 30 g/L of magnesium carbonate. Lactic acid production was further enhanced by using immobilized cells with coconut shell-activated carbon (CSAC) of different sizes (A, B, C, and D) in the repeated batch cultivation of dual-phase fermentation producing 31.64 g/L of lactic acid. This work sheds light on the possibilities to enhance the utilization of PKC for lactic acid production via immobilized *A. succinogenes* 130Z.

## Introduction

The explosion of industrialization caused by the world's growing population has increased demand for petroleum-based materials, which could lead to depleting reserves, rising prices, and the unfolding energy crisis. This phenomenon verifies the necessity of alternatively utilizing renewable resources (i.e. biomass) and cleaner processing pathways (i.e. fermentation system) to promote energy sustainability, economic efficiency, and eco-friendliness with lesser environmental impacts^1^. In this regard, PKC could be a viable alternative for the bio-conversion of industrially important compounds like lactic acid given its plentiful, renewable, and inexpensive source of biomass. The potential of PKC as a sustainable feedstock has caused a great deal of interest due to its comparable amount of fermentable sugars to other potential oil palm biomass sources like empty fruit bunches (EFB) and oil palm trunk bagasse (OPTB). These materials had previously been used to produce lactic acid successfully^2–4^, and PKC is seen as a promising alternative feedstock for the same purpose^5^. One of the key advantages of PKC is that it contains a majority of the macro- and micronutrients required for a basal fermentation medium^6^, making it a valuable resource for sustainable bio-based fine chemical production. Moreover, PKC has low levels of lignins and their derivatives, making it a clean material that can be readily hydrolyzed with only mild pretreatment. This process generates negligible amounts of inhibitory byproducts.

With almost 70% of carbohydrates composed of fermentable sugars, the abundantly available lignocellulosic biomass can be converted into second-generation biofuels and chemicals^2^. In order to maximize the advantageous nutrient content of PKC and create multi-functional value-added products while fostering a circular economy and sustainability, as well as increasing national income, the Malaysian government has set aside a budget of approximately US$ 15 million for research and development (R&D) related to oil palm biomass utilization. In 2019, 2.59 million tons of PKC were produced annually out of ca. 80 million tons of agricultural biomass, and is expected to increase further in the coming years. However, the PKC remains poorly exploited since it is only marginally used to make feed additives for livestock, with low nutritional and amino acid levels, poor protein digestibility, and a rough texture causing remnants to degrade on the ground and pollute the environment. Feed additives represent the second most economically valuable category of bioproducts after fine chemicals, and are followed by bioadhesives, biobutanol, biohydrogen, and biofuel^11^. However, the PKC bio-products with the highest economic value have not been given as much attention. Therefore, investigating the potential of PKC as a feedstock for a fine biochemical with a higher economic value, such as lactic acid, could be a novel way to maximize the advantageous nutrient content of PKC, while also promoting the usage of locally-harvested biomass, fostering a circular economy and sustainability, and increasing national income. Therefore, research is being conducted to develop multi-functional value-added products from this renewable and readily accessible biomass. Lactic acid can be produced anaerobically through the use of homolactic microorganisms and modified strains of lactic acid-producing bacteria^2^. This study represents a significant breakthrough in lactic acid production, as it employs a novel approach utilizing the facultative anaerobic strain of *A. succinogenes* 130Z in a dual-phase (aerobic-anaerobic) fermentation involving both glucose and mannose. This innovative approach resulted in a remarkable 22-fold increase in productivity compared to traditional anaerobic conditions^4^. The versatility of *A. succinogenes* 130Z could be exploited to achieve a more efficient conversion of the available sugars, resulting in a significantly higher yield of lactic acid.

The potential of sugar recovery in PKC has been studied using a number of different pretreatment techniques. Several studies found that dilute acid hydrolysis yielded satisfactory sugar recoveries of 33.1–37.0 g/L from PKC, whereas hydrothermally pretreated PKC yielded 28.34–71.54 g/L of sugars^5,6^. The studies further proved the fermentability of PKC for the production of previous high-value bio-products such as bio-hydrogen, bioethanol, prebiotic, poultry diet feed additives, bio-adhesive, and biofuels. Hence, PKC can be employed as one of the most promising feedstocks for the synthesis of lactic acid, which is known with a long list of uses in the production of food, cosmetics, medicines, secondary conversion chemicals, and other common chemicals^7^. Over than half of the lactic acid produced worldwide is focused primarily on food industry as an acidulant, preservative, food flavoring, emulsifying agent, and pH agent in several food products, including confectioneries (such as sweet, and chocolate), dairy products (such as yoghurt, and cheese), beverages (such as beer, wine, and soft drinks), bakery goods, and pickled foods^8^. Thus, biological manufacture using renewable resources has gained a lot of recognition.

As with other lignocellulosic biomass, PKC is mostly composed of cellulose, hemicellulose, and lignin^9^. A key element in maximizing sugar recovery from the lignocellulose structure is enzymatic saccharification, where holocellulosic polymers are selectively converted into monomeric sugars with high purity^10^. However, the PKC is very resilient to enzymatic breakdown for saccharification and fermentation due to its recalcitrant lignocellulosic composition^11^. Therefore, the use of an appropriate pretreatment technique is required to break down the structural carbohydrate components of PKC in the form of cellulose and hemicellulose^12^. In addition to cellulose, using hemicellulosic fraction in PKC is to maximize the carbon source utilization for subsequent fermentation.

Alkaline pretreatment is a potential method to increase the surface area of carbohydrates available for enzymatic digestion. However, the formation of phenol, an inhibitory byproduct, necessitates a detoxification step that adds to the overall cost of the pretreatment process of PKC. Hemicellulose can be hydrolyzed through dilute acid pretreatment, which typically employs hydrochloric acid, nitric acid, phosphoric acid, or sulfuric acid^13^. Studies have found that this method of lignocellulosic biomass pretreatment yields high recovery of monomeric sugars and increased enzymatic digestibility of hemicellulose. Nevertheless, mineral acids are less favorable due to drawbacks such as high levels of gypsum production, which hinders downstream processes and corrodes equipment, as well as inhibitory byproduct accumulation from sugar and lignin breakdown^12^. A prior study which compared alkali, dilute acid, and auto-hydrolysis pretreatments on PKC revealed that the highest inhibitory products, i.e., formic acid, furfural, and HMF, were present in both severe sulfuric acid and sodium hydroxide pretreatments^14^. The use of dilute oxalic acid pretreatment has been suggested as an alternative due to its efficacy in breaking down hemicellulose into monomeric sugars at relatively low concentrations, its non-inhibitory effect on glycolysis, its lower toxicity to microorganisms compared to sulfuric and acetic acids, its odorlessness, and its cost-effectiveness^15^. Additionally, HMF and furfural, as inhibitory compounds, have been identified as trace elements in hydrolysates pretreated with oxalic acids^16^. A similar study was conducted to assess the effectiveness of oxalic acid, formic acid, and citric acid for the hydrolysis of OPTB with xylose recoveries of 61.2%, 20.1%, and 12.9%, respectively. The superior performance of oxalic acid is attributed to its dicarboxylic structure, which is capable of selectively hydrolyzing hemicellulose to yield high monomer sugars and a solid with high enzymatic saccharification digestibility^15–17^. Therefore, it is expected that dilute oxalic acid will be effective for pretreatment of PKC with comparable levels of sugars to OPTB.

Fermentable sugars liberated from the pretreated PKC can be converted to bio-based lactic acid primarily by several types of anaerobic microorganisms such as lactic acid bacteria (LAB), *Corynebacterium glutamicum*, *Bacillus coagulans, and Escherichia coli*^18–20^. Anaerobes are, nevertheless, economically unfavorable due to the need for the specialized equipment, medium, and expertise for cultivation^18^. Therefore, using facultative anaerobic *A. succinogenes* 130Z in an aerobic-anaerobic technique of dual-phase fermentation was found to be 22 times higher productivity than in typical anaerobic conditions^4^. *A. succinogenes* 130Z was selected as the organism for lactic acid production due to several reasons. Firstly, it is categorized as a biosafety level 1 (BSL-1) organism, which is generally regarded as safe and non-pathogenic by the DSMZ and ATCC^21^. *Secondly, A. succinogenes* has the ability to utilize a broad range of carbon sources, including glucose, xylose, arabinose, mannose, galactose, fructose, lactose, cellobiose, mannitol, maltose, glycerol, and sucrose, in anaerobic environments due to its natural ecological habitat in the bovine rumen^22^. This offers many different carbon sources for fermentation, which is a major advantage. Finally, *A. succinogenes* is known to be a strong candidate for producing succinate and lactate due to its ability to tolerate high organic acid concentrations and other potential growth inhibitors^19,21,22^. As for the two-phase process mode, it was found that immobilization of *A. succinogenes* 130Z could maximize the sugars usage and productivity of lactic acid from pretreated biomass.

Nevertheless, the fermentation of free *A.succinogenes* 130Z cells in a mixed carbon sources from PKC hydrolysate may cause hemicellulose catabolic repression^24^. Alternative strategies have been put forth including immobilization system which enables cell-entrapment in a feasible condition, hence promoting high cell aggregation, cell density and substrate consumption^25^. At the same time, cell entrapment may induce cell-transfer resistance due to the limitation of nutrient diffusion through the support matrix. The demerit can be overcome by selecting a good support material for cell adhesion as a key success for the immobilization process. Coconut shell-activated carbon (CSAC) is the potential support material with the criteria as inert, cost-effective, has a greater mechanical strength, good wear resistance, higher surface area and porosity due to the internal granular structure than any other activated carbons of a similar grade^26^. The determining factor for an excellent support material is to have a high specific surface area, resulting in high productivity of lactic acid^26^. Therefore, the different sizes of specific surface area of CSAC were compared to develop an industrially feasible fermentation using PKC hydrolysate.

The primary goal of this study was to evaluate the fermentability of the resultant oxalic acid pretreated PKC hydrolysates and fermentation method i.e. immobilized cells for high recovery of fermentable sugars from PKC and its effects on fermentability performance by *A. succinogenes* 130Z in the production of lactic acid. The effects of oxalic acid concentrations (1–6% w/v) and residence times (1–5 h) on the sugar recovery were initially investigated followed by the enzymatic saccharification of the pretreated PKC. Subsequently, the fermentability performance of PKC hydrolysate was evaluated and compared in terms of cultivation phases (i.e. mono and dual-phases), carbonate loadings (i.e. magnesium and sodium carbonates), and types of sugars (i.e. glucose and mannose). Lactic acid production was further enhanced by using immobilized cells with coconut shell-activated carbon (CSAC) of different sizes (A, B, C, and D) in the repeated batch cultivation of dual-phase fermentation.

## Results and discussion

### The effect of residence time and oxalic acid concentration on fermentable sugar production

**Figure 1:**
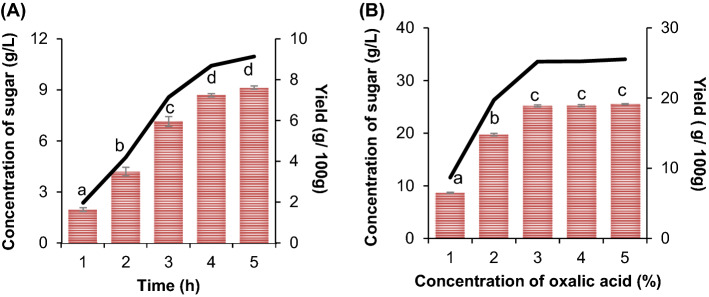
The effect of (**A**) residence time (1–5 hs) and (**B**) concentration of oxalic acid (1–5%). Note: a–d represent a grouping information through Tukey’s Method at 95% confidence. Means that do not share a same letter are significantly different; data presented are calculated as means ± standard deviations of triplicate runs.

Accurate compositional analysis of biomass allows for the evaluation of conversion yields and process economics^27^. Analysis conducted for raw PKC showed that the contents of glucan, mannan and lignin were 10%, 33.1% and 20.6%, respectively. Following that, pretreatment using oxalic acid was performed at different concentrations (1–5%, w/v) and reaction times (1–5 h) to find a suitable catalyst conditions in hydrolysing the PKC, aiming at recovering the highest possible amount of fermentable monomeric sugars in the form of mannose and glucose. Accordingly, oxalic acid affects the composition of PKC components by hydrolysing the hemicellulose component (releasing mostly mannose in the pretreated liquor)^28^. During the acid pretreatment, the sugar released was higher at higher oxalic acid concentrations under the severity factor of log R_0_ = 0.684. The interaction between residence time and acid concentration on mannose accumulation was investigated using one-factor-at-a-time (OFAT) approach as depicted in Fig. 1.

The effect of residence time on hydrolysis was studied by maintaining the temperature at 121 °C and the concentration of 1% oxalic acid. As could be seen in Fig. 1A, the long residence time was demonstrated to elevate the production of sugar, as it could extend more efficient solubilization of the hemicellulose fraction of PKC. The hydrolysis lasting for 4 h yielded the best sugar concentration of up to 8.68 g/L with 21.4% mannose recovery; conversely, the hydrolysis lasting beyond this period are no longer significant to increase the production of fermentable sugars. A maximum mannose recovery of 62.6% was achieved using 3% (w/v) oxalic acid as compared to 21.7–49.1% at lower oxalic acid concentrations of 1–2% (w/v) (Fig. 1B). When the acid concentrations were used at 4–5% (v/v), the concentration of sugars levelled off. The influence of acid concentration was greater than reaction time in this regard, which indicated that a higher oxalic acid concentration is required to efficiently hydrolyse hemicellulose from the PKC. Therefore, the optimum residence time and oxalic acid concentration were found to be 4 hs and 3% (v/v) oxalic acid, respectively.

On the other hand, cellulose was preserved at > 95% (w/v) in the pretreated PKC at various oxalic acid concentrations, indicating that the cellulose was not easily degraded to monomeric sugar by oxalic acid catalyst under the mild conditions employed. The yields of mannose, glucose and acetic acid obtained following oxalic acid pretreatment are shown in Table 1. As the amount of recovered mannose was higher than glucose when PKC was pretreated with oxalic acid in all cases, oxalic acid was determined to be effective in hydrolyzing hemicellulose into monomeric sugar, i.e. mannose. Similarly, Bukhari and co-workers obtained higher yield of hemicellulose i.e. xylose (60.3%) while pretreating OPTB with 4% (w/v) oxalic acid at 121 °C for 100 min^28^. On a different note, about 8.91 g/L mannose was obtained from PKC pretreated using 1% (v/v) sulfuric acid^29^ and 13.54 g/L mannose was obtained from acai seeds pretreated using 1.5% (w/v) sulfuric acid at 121 °C^30^; both of which are comparable to the findings in this study.

**Table 1:** Mannose, glucose and acetic acid released from pretreated PKC at different oxalic acid concentration.

Oxalic acid concentration (%, w/v)	1	2	3	4	5
Mannose (g/ 100 g)	8.7 ± 0.1	19.7 ± 0.25	25.1 ± 0.2	25.2 ± 0.2	25.5 ± 0.1
Glucose (g/100 g)	0.05 ± 0.01	0.22 ± 0.05	0.39 ± 0.02	0.52 ± 0.09	0.5 ± 0.19
Acetic acid (g/100 g)	0.01 ± 0.005	0.029 ± 0.001	0.031 ± 0.001	0.04 ± 0.001	0.053 ± 0.001

Hemicellulose is amorphous and requires less severe digestion condition for xylose to be released during pretreatment compared to the majorly crystalline cellulose counterpart^31^. The amorphous region of cellulose is more accessible and can be broken down into glucose monomers during acid pretreatment. However, glucose was found at much lower concentration of around 0.05 to 0.5 g/L during the pretreatment of PKC with different oxalic acid concentrations.

Moreover, acetic acid, an inhibitory compound, was accumulated at higher oxalic acid concentration (Table 1). The severity factor for the optimum oxalic acid hydrolysis used in this study was calculated according to the equation described by Bukhari et al.^28^ and was determined as 0.684, which is comparatively low. The released acetic acid concentration was 0.031 g/L when pretreated with 3% (w/v) oxalic acid. The acetyl groups on the side chain of hemicellulose are responsible for this phenomenon as they are mostly susceptible to be hydrolysed to acetic acid under more severe conditions^17^. It was reported that negligible amounts of formic acid, furfural and HMF were detected in oxalic acid-pretreated hyrolysate of PKC due to low severity employed (i.e. log R_0_ < 1.0)^28^. The formations of formic acid, furfural and HMF were not detected in this study, which further proved the mild conditions established for oxalic acid pretreatment. However, oxalic acid pretreatment is insufficient to fully unlock the maximum fermentable sugar recovery from the PKC. Hence, enzymatic hydrolysis is needed to further digest the cellulose fraction of the PKC.

### Enzymatic hydrolysis of pretreated PKC

**Figure 2:**
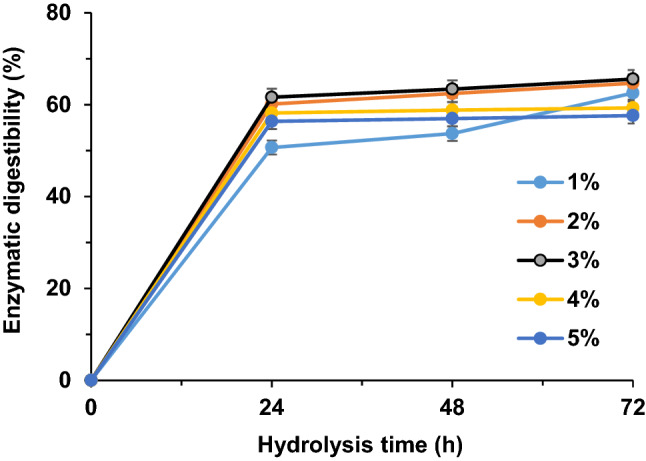
Enzymatic digestibility of pretreated PKC using different oxalic acid concentration.

The cellulose saccharification of pretreated PKC were conducted with 72 h of enzymatic hydrolysis. The pretreated PKC with 3% (w/v) oxalic acid demonstrably had the highest enzymatic digestibility of 65.6% (Fig. 2), and correspondingly exhibited the highest glucose yield of 9.14% g/g (Table 2).

**Table 2:** Sugar released after oxalic acid pretreatment followed by enzymatic hydrolysis at 72 h.

Oxalic acid concentration (%, w/v)	1	2	3	4	5
Enzymatic digestibility (%)	62.5 ± 0.1	64.7 ± 0.15	65.6 ± 0.1	59.3 ± 0.2	57.6 ± 0.1
Mannose (g/100 g)	8.68 ± 0.1	19.7 ± 0.25	25.18 ± 0.2	25.23 ± 0.2	25.5 ± 0.1
Glucose (g/100 g)	8.38 ± 0.1	8.85 ± 0.05	9.14 ± 0.1	8.42 ± 0.1	8.18 ± 0.2
Total sugar (g/100 g)	17.06 ± 0.2	28.55 ± 0.3	34.32 ± 0.3	33.65 ± 0.3	33.68 ± 0.3

This glucose yield was higher than that obtained from corncob pretreated at much higher concentration of oxalic acid (15%, w/v), i.e., 25.18% of fermentable sugars^32^. Conversely, pretreatment using 5%, w/v of citric acid and acetic acid only recover 12.9% and 20% of sugars, respectively^28^. Meanwhile, at lower oxalic acid concentrations (1–2%, w/v), the enzymatic digestibility was at lower rate of 64.7%. These findings showed that 3% (w/v) oxalic acid pretreatment was able to provide a maximum accessibility of PKC to enzymatic digestion. Greater hemicellulose degradation generally led to higher rates of cellulose-to-glucose conversion as the degraded hemicellulose tends to create a cross-linked network for structural integrity of cell walls by binding to cellulose microfibrils and lignins.

### Morphology of PKC structures

**Figure 3:**
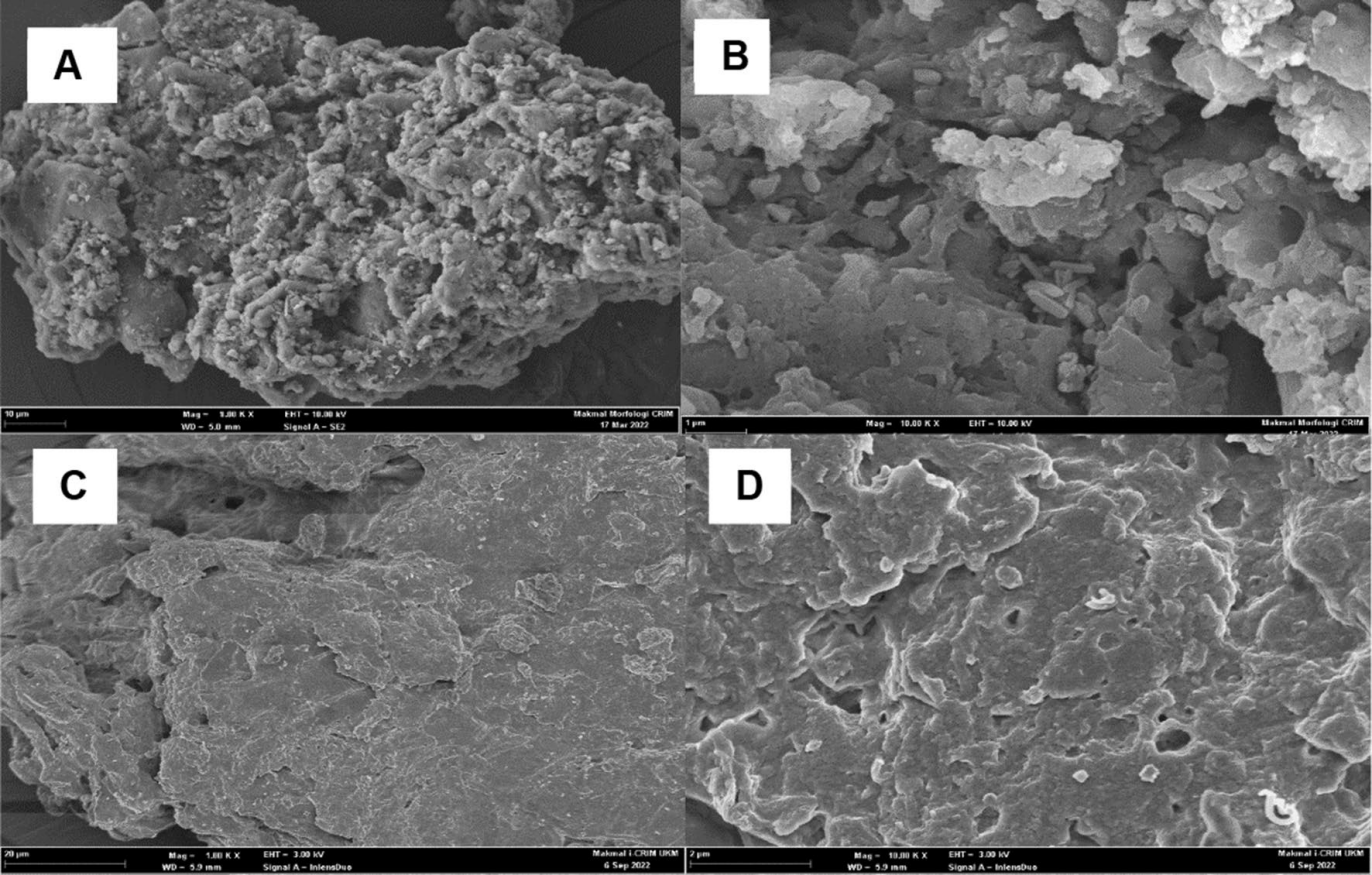
FESEM image of (**A**) cross-sectional of untreated PKC at × 1.00 k magnification (**B**) cross-sectional of untreated PKC at × 10.00 k magnification (**C**) cross-sectional view of pretreated PKC with 3% (w/v) Oxalic acid at 121 °C for 4 hs at × 1.00 k magnification (**D**) pretreated PKC at × 10.00 k magnification.

Field emission scanning electron microscope (FESEM) analysis of the morphological structures of untreated and treated PKC samples is shown in Fig. 3. The oxalic acid pretreatment has roughened the PKC surfaces (Fig. 3D). Oxalic acid breaks down natural polymers into their basic monomers by de-bonding and cracking of lignocellulose-matrix interface causing the lignocellulose surface to be disrupted and altered. Figure 3A demonstrates that the surface structure of untreated PKC has a more compact and intact surface than the pretreated PKC^33^, which has a rough and distorted surface^34^. These findings also point to the removal of extractives and hemicelluloses during the hydrolysis while leaving the insoluble lignin unaltered. The breakdown of the crystalline structure of the treated PKC, which eliminated the amorphous components of the biomass, caused the structural alterations in the cell wall^35^.

### X-Ray Diffraction of PKC structures

**Figure 4:**
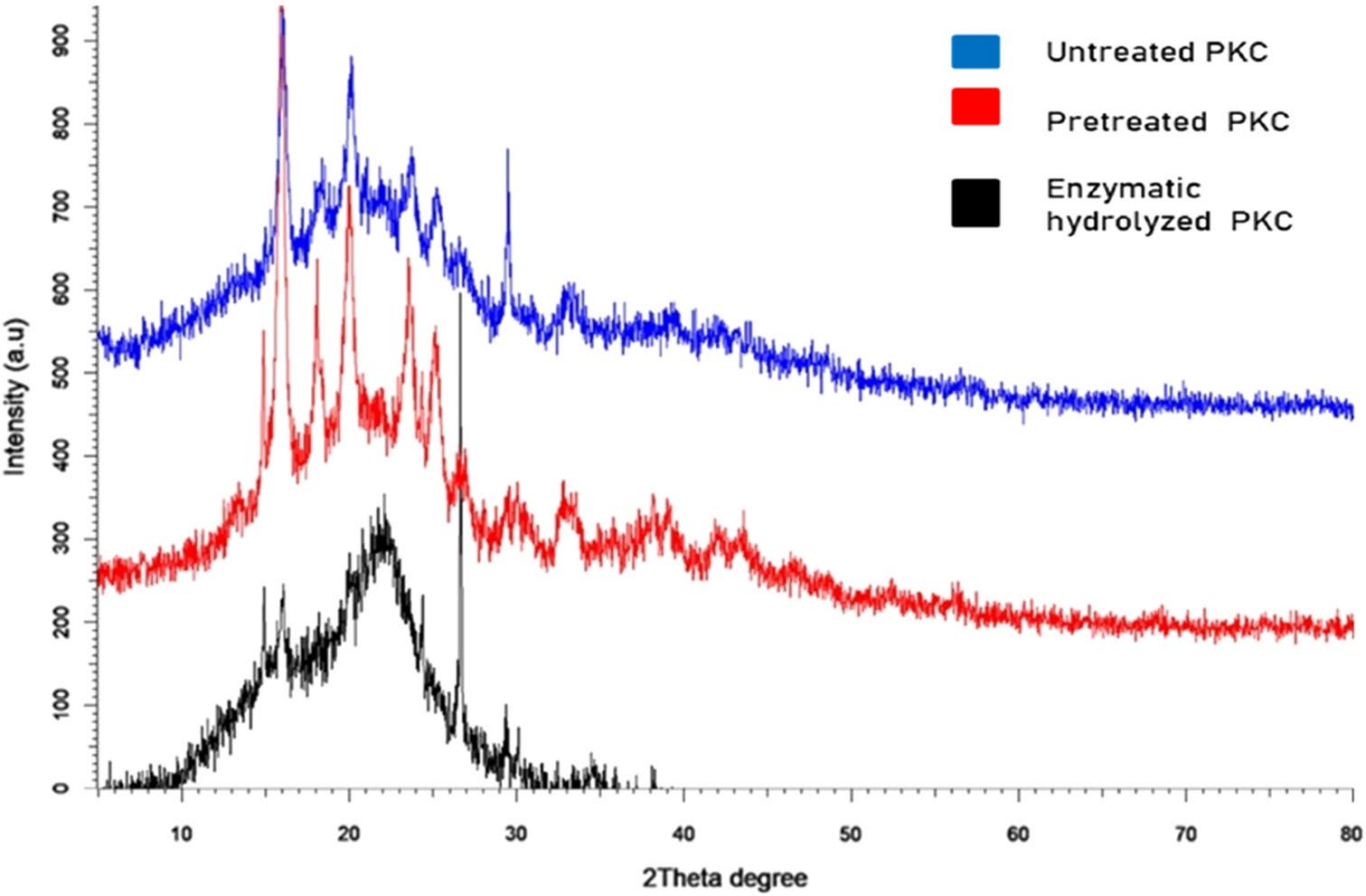
X-ray diffraction patterns for untreated and treated PKC with oxalic acid pretreatment and enzymatic hydrolysis.

An X-Ray Diffraction (XRD) characterization was used to compare the crystallinity of cellulose in PKC before and after hydrolysis as seen in Fig. 4. The crystallinity index (CrI) indicates the proportion of crystalline cellulose in the total solid of biomass. It was discovered that the CrI of raw PKC was around 26.3%. Following oxalic acid hydrolysis, it seems that the CrI value increased to 35.9%. The hydrolysis has successfully removed the non-cellulosic components from PKC that were present in the amorphous areas resulted in an increase in the magnitudes of these crystalline peaks. The hydrolysis process exposes more cellulose on the fibre surface, thereby increasing the roughness of the surface. Then, CrI value went down to 23.3% after removal of cellulose in enzymatic hydrolysis process, indicating the increase in lignocellulosic digestibility. These findings demonstrated that the treated PKC with 3% (w/v) oxalic acid at 121 °C for 4 hs was able to provide a maximum PKC slurry accessible to enzymatic digestion.

### Comparative effect of mono-phase and dual-phase fermentation on lactic acid production.

**Figure 5:**
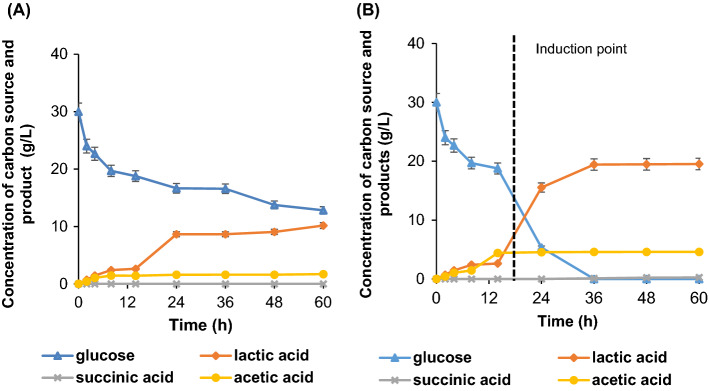
The time-course profile of batch fermentation of lactic acid production by *A.succinogenes* 130Z in (**A**) mono-phase cultivation (**B**) dual-phase cultivation.

The oxalic acid-pretreated PKC hydrolysates was further assessed to evaluate the fermentability test of PKC hydrolysate on the productivity of lactic acid. Facultative anaerobic *A. succinogenes* grows well in an anaerobic cultivation to produce succinic acid in its indigenous metabolic pathway but can be adapted to lactic acid producing pathway by providing anaerobic–aerobic dual-phase conditions^36^. Oxygen level is detected by the cell metabolic system, generating signal ready for the gene expression modification^37^. Hence, oxygen level in different cultivation modes has a remarkable effect on the organic acids production. In this study, different phases of cultivation were tested for the production of lactic acid using the *Actinobacillus succinogenes*. Mono-phase anaerobic fermentation and dual-phase fermentation were compared in the 250 mL flask containing 100 mL fermentation medium. For the mono-phase anaerobic cultivation depicted in Fig. 5A, lactic acid produced at the titer of 10.16 g/L with an overall yield of 0.59 g/g. Succinic acid was not detected and the glucose consumption was slow. While, the gradient of cell growth rate and glucose consumption spiked higher in the dual-phase cultivation after the induction with MgCO_3_ at 14 h (denoted as induction point) as shown in Fig. 5B. As a result, lactic acid was produced at the higher titers of 19.4 g/L with an overall yield of 0.65 g/g. These results indicate that the *A. succinogenes* yielded high titer of lactic acid under appropriate strategy.

The dual-phase operation promotes approximately double increment of lactic acid production as compared to mono-phase anaerobic fermentation. This production trends were in agreement with the previous findings by Li et al., where dual-phase cultivation afforded 22-fold higher lactic acid than mono-phase cultivation^4^ It was reported that the dual-phase cultivation has correlation with higher lactic acid yield as the initial oxygen introduction promotes higher cell growth rate, improve cellular viability and facilitate the shift of C4 to C3 flux tremendously in the first phase, while inducing high yield in the latter phase^38,39^. The lactate-producing C3 flux key driver, 6-lactate dehydrogenase (encoded LDH) was also reported to produce approximately 18-fold higher in dual phase cultivation than in mono-phase anaerobic cultivation. Therefore, lactic acid titer, yield and productivity can be significantly improved in the later anaerobic cultivation with no genetic modification required. The findings were remarkably significant to substitute cell genetic modifications known with few limitations i.e. metabolic imbalance due to nutrition deprivation, metabolite buildup, evolutionary pressure, plasmid instability, and other stress factors^40^.

### Effects of carbonate loading on lactic acid production

In this study, different carbonate salt using magnesium carbonate and sodium carbonate was added into the fermentation media at different loadings to investigate the synergistic effect of dissolved carbon dioxide and continuous pH neutralizing agent on the lactic acid production from PKC hydrolysate by *A. succinogenes.* pH level is a critical factor in the regulation of intracellular enzyme activities and cellular viability for the optimum performance of bacterial cultures. Hence, the use of neutralizing agents is required to prevent the medium from becoming too acidic with the continuous formation of other organic acid byproducts, i.e. succinic, formic and acetic acids, during fermentation^41^. Furthermore, a study found that dissolved CO_2_ was not sufficient enough to optimize organic acid formation when gaseous CO_2_ was used as a sole inorganic carbon source donor in the fermentation of *A. succinogenes* ATCC 55,618^42^. Therefore, the supplementation of carbonate salt for CO_2_ fixation within the cells is required to keep the reaction in equillibrium by replacing the used up gaseous CO_2_ in the fermentation, hence promoting a maximum lactic acid yield^43^.

**Figure 6:**
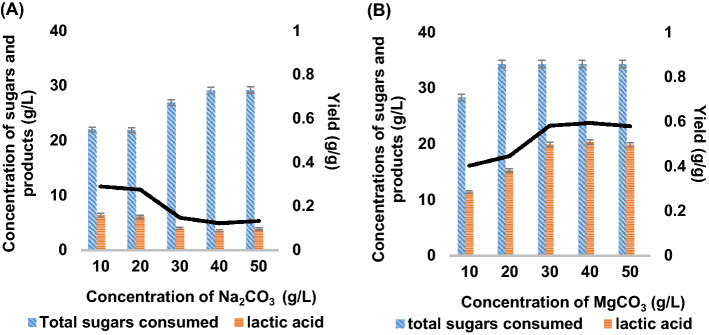
Lactic acid concentration and sugar consumption by *A. succinogenes* at different carbonate loading with (**A**) Na_2_CO_3_ loading, and (**B**) MgCO_3_ loading.

**Figure 7:**
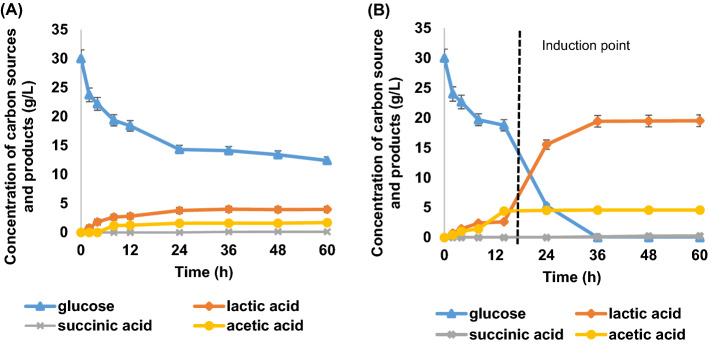
Lactic acid concentration and sugar consumption by *A. succinogenes* at different carbonate loading with (**A**) 20 g/L of Na_2_CO_3_ loading, and (**B**) 30 g/L of MgCO_3_ loading.

Figure 6 showed the final concentration of lactic acid and total sugar consumption from the PKC hydrolysate after 60 h by using different levels of carbonate salts loading from 10 to 50 g/L. As illustrated in Fig. 6A, the production of lactic acid with Na_2_CO_3_ was deplorable, getting decreased with higher Na_2_CO_3_ loading resulting in less than 6.4 g/L of lactic acid after 60 h under anaerobic fermentation. Although Na^+^ promotes cellular nutrient intake and regulates intracellular pH, higher Na^+^ loading can suppress cellular growth with the accumulation of hyperosmotic stress and cell flocculation resulting in low lactic acid production as demonstrated in Fig. 7A ^44^.

Conversely, fermentation with MgCO_3_ resulted in the significantly high lactic acid accumulation. As seen in Fig. 6B, the highest concentration obtained was 28.9 g/L with the supplementation of 30 g/L of MgCO_3_. Lactic acid production increased steadily from 10 to 30 g/L of MgCO_3_ after which lactic acid production was levelled off. The increase in carbonate salt loading (CO_2_ supplier) yielded higher lactic acid in *A. succinogenes* from PKC hydrolysate. The findings were in agreement with previous findings by Li et al.^45^ who reported that MgCO_3_ is the best neutralizing agent than Na_2_CO_3_, NaHCO_3_, Mg(OH)_2_, Ca(OH)_2_, CaCO_3_, NaOH and NH_3_·H_2_O in terms of cell growth, glucose consumption, succinic acid formation and yield of glucose-to-succinic acid conversion in *A. succinogenes* NJ113. Furthermore, Mg^2+^ does not impede cellular membrane stability and no cell flocculation was seen, hence the cell growth and production of lactic acid remain stable^46^.

MgCO_3_, in the form of carbonate salt, is recognized as an effective pH regulator for microbial fermentation. It provides a continuous supply of CO_2_, which is essential for microbial growth, and Mg^2+^ ions, which act as cofactors for various enzymatic reactions within the cells. One of the critical enzymes in the microbial metabolism is phosphoenolpyruvate carboxykinase (PEPCK), which catalyzes the conversion of oxaloacetate into phosphoenolpyruvate (PEP) in the first step of the TCA cycle. PEP is then converted into pyruvate through lactate-forming C3 pathway or oxaloacetate in the succinate-forming C4 pathway. Herein, the presence of Mg^2+^ ions enhances the activity of PEPCK, leading to an increased conversion of oxaloacetate into PEP, which can then be utilized in either the C3 or C4 pathways. As a result, the continuous supply of Mg^2+^ ions through MgCO_3_ helps to maintain a stable pH and promote optimal metabolic activity in the fermentation process^47^. On another note, the incomplete TCA cycle of *A. succinogenes* can be manipulated by introducing initial aeration of microaerobic cultivation followed by introducing MgCO_3_ as CO_2_ supplier in anaerobic condition to significantly stimulate lactate-forming C3 pathway^36^. This is evidenced in Fig. 7B, where the addition of MgCO_3_ at 14 hs after initial aeration significantly increases sugar consumption and lactate formation due to its effectiveness as continuous pH regulator. The finding is also in agreement with the previous report that MgCO_3_ is also an efficient buffer factor for the succinate production^44^.

### Effects of individual sugar on lactic acid production

**Figure 8:**
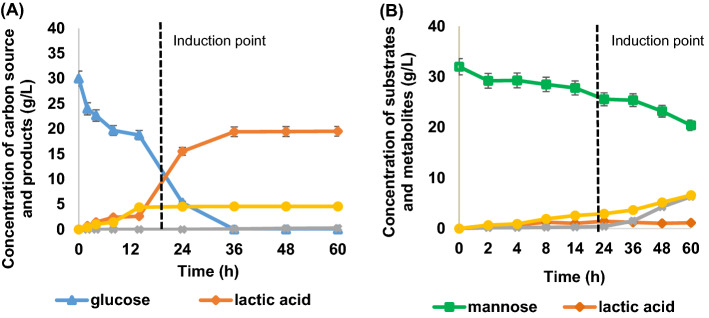
The time-course profile of batch fermentation of lactic acid production with substrate (**A**) glucose and (**B**) mannose.

To our knowledge, no study has examined lactic acid production by *A. succinogenes* using the most prevalent monosaccharides present in the PKC, i.e., glucose, and mannose. For this reason, lactic acid production from different monosaccharides were assessed in batch fermentations. These sugars are all present in PKC hydrolysate. The batch fermentation were performed by enriching the standard media with the studied commercial glucose and mannose as carbon feedstock. Figure 8 shows that *A. succinogenes* was able to convert the investigated sugars and the conversion degree were dependent on the sugar metabolizability. Figure 8 shows the time-course profile of batch fermentation of lactic acid production with glucose and mannose as carbon sources.

Based on the trend, the glucose consumption was directly proportional to the production of lactic acid. At 24 h, the glucose consumption rate decreased and the lactic acid concentrations nearly levelled off. The fermentation was further continued for up to 60 h, wherein glucose conversion went up to 100% and lactic acid was constantly yielding up to 19.4 g/L. The fermentation was completed after 24 h when the substrate were depleted and metabolites were constant. However, the mannose fermentation with initial concentration of 30 g/L ceased when lactic acid concentration approached a value of 1.21 g/L despite of incomplete mannose-lactic acid conversion as depicted in Fig. 8B. The productivity of lactic acid yield significantly lower as compared to glucose. The observed trend in the mannose fermentation profile was congruent with that reported in the literature^48,49^. However, no clear explanation available in the previous literature regarding such a lower performance of mannose than glucose utilization. The difference in transport system of mannose than glucose may explain the difference in yield and productivity of lactic acid obtained. Meanwhile, glucose was confirmed to be the preferred sugar, with the overall performance consistent with previous findings^48^.

### Fermentability test of PKC hydrolysate on lactic acid production

**Figure 9:**
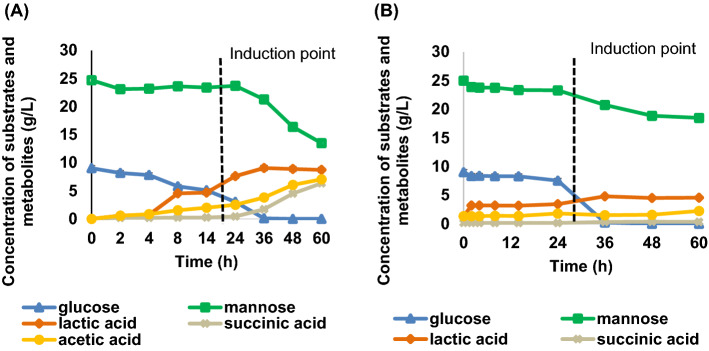
The time-course profile of batch fermentation of lactic acid production in (**A**) model hydrolysate and (**B**) PKC hydrolysate.

The fermentability of PKC hydrolysate was evaluated to examine the profile of lactic acid formation by *A. succinogenes* 130Z with its by-products formation (succinic acid, formic acid and acetic acid), as well as the sugars uptake (mannose and glucose) (see Fig. 9). We examined the fermentation trends of model hydrolysate (Fig. 9A) and PKC hydrolysate (Fig. 9B), wherein lactic acid accumuation in the PKC hydrolysate reached a maximum concentration of 4.79 g/L in less than 24 h (Fig. 9B). Within the first 24 h of fermentation, there was a huge drop in sugar level that was followed by a surge of product formation as shown in Fig. 9B.

Glucose which was present in relatively smaller amounts than mannose was exhausted after 36 h. Meanwhile, mannose was not completely consumed within 60 h, and mannose uptake did not appear to begin until glucose was mostly consumed. However, once glucose supply was exhausted, the mannose was readily consumed. The trend was in agreement with the previous finding which performed batch fermentation of *A. succinogenes* with mixed sugars^48^. As in the case of PKC hydrolysate, a decreased yield (0.37 g/g vs. 0.71 g/g) and productivity (0.13 g/L/h vs. 0.25 g/L/h) were attained, with a 47% lower lactic acid content than the reference model hydrolysate. The low lactic acid productivity in the PKC hydrolysate was caused by the inhibition in lactic acid production by *A. succinogenes.* The possible explanation for the phenomenon was due to the presence of high concentration of oxalate salts in the pretreated PKC hydrolysates produced from the neutralization process of the hydrolysate. It was reported that bacterial cells were unable to tolerate with 2–4% of oxalic acid concentration^28^.

### Production of lactic acid with immobilized cells

#### Bacterial observation on coconut shell-activated carbon

The use of the cells immobilisation technique was justified by the high cell retention, which would improve the uptake of lignocellulose sugars. Therefore, the high sugar uptake in this case promote higher lactic acid production. According to a study by Indera Luthfi and co-workers, CSAC is the best support carrier for *A. succinogenes* as it promotes high cell concentration compared to exclay, kieselguhr, and vermiculite owing to its greater specific surface area, well pore distribution, and higher EPS content^50^. Good surface area provides large capacity for cell retention, and promotes high EPS content, which enhances irreversible cell attachment, resulting in improved lactic acid production. Emerging data has shown that *A. succinogenes* cells may adhere naturally to the surfaces of a solid support carrier and inevitably establish a biofilm when processes are carried out over an extended period of time^51^.

**Figure 10:**
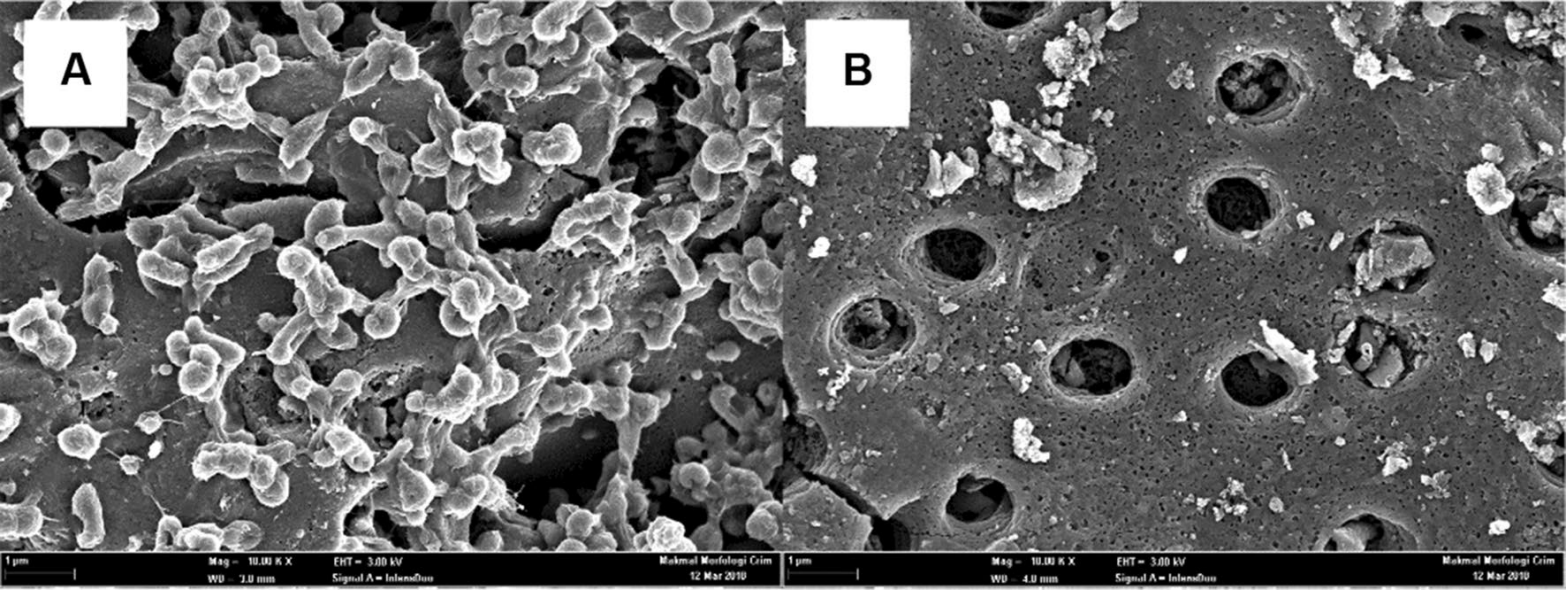
FESEM image of (**A**) cells immobilized on CSAC at × 10.00 k magnification (**B**) (CSAC) at × 10.00 k magnification.

Colonies of *A. succinogenes* 130Z were found to grow inside the pores of CSAC after repeated batch cultivation of 180 h and were observed under FESEM as shown in Fig. 10A. Coccobacilli-shaped *A. succinogenes* cells that existed in clumps and were intermingled were seen in Fig. 10A. Additionally, it was demonstrated that the CSAC had a very porous structure that afforded capacity for cell immobilization as shown in Fig. 10B.

#### Repeated batch fermentations using immobilized cells with different sizes of coconut shell-activated carbon

**Figure 11:**
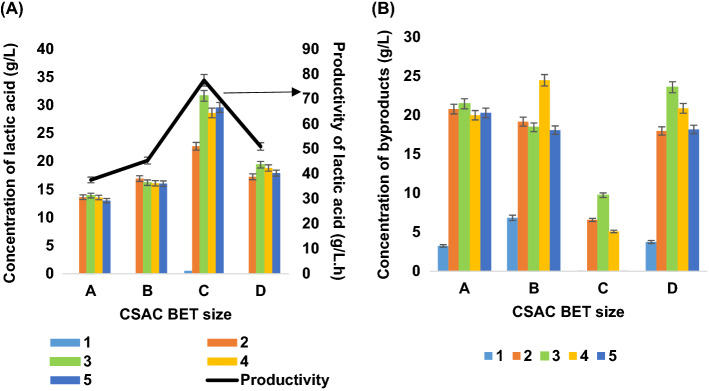
Fermentation of immobilized *A. succinogenes* with different sizes of CSAC (**A**) Concentration of lactic acid (**B**) Concentration of byproducts.

The purpose of the repeated batch fermentation in this study was to determine the effect of CSAC size on the performance of cell immobilisation. Figure 11A showed the final lactic acid productivity for several repeated batches over a period of around 180 h using coconut shell-activated carbon (CSAC) as the support carrier. The inferior performance of lactic acid productivity for all CSAC sizes were observed within the first 12 h. The possible explanation for the behavior is due to the microbial adaptation phase to the environment. A higher and more stable trend in lactic acid productivity for all CSAC sizes were demonstrated from batches 2 to 5 within a significantly short period of time, indicating that the cells had well-familiarized with their environment^50^.

In this work, CSAC C afforded the best lactic acid productivity of 0.88 g/L/h (Fig. 11A) compared to D, followed by B, and A with 0.51 g/L/h, 0.45 g/L/h and 0.38 g/L/h, respectively. The performance of an immobilization carrier depends on the size of specific surface area and total pore volume of CSAC. Both of adhesion capacity of the cell binding to the surface and cohesion capacity of the cells interaction with other cells corresponded to the surface area and pore properties of carriers^51^. Table 3 outlines the properties of the different sizes of CSAC in this work. As could be seen in Table 3, the larger pore volume demonstrated to elevate the productivity of lactic acid as it could extend higher capacity of cell density. The CSAC C demonstrated the best lactic acid productivity of up to 0.88 g/L/h; conversely, the larger CSAC size of pore volume beyond this size are no longer significant to increase lactic acid productivity. A maximum lactic acid productivity of 0.88 g/L/h was achieved using CSAC C as compared to 0.38–0.45 g/L/h using smaller sizes (Fig. 11A). The findings proved that CSAC-immobilized cells were able to improve lactic acid production from PKC hydrolysate by a concentration of 31.64 g/L and 0.92 (g/g) yield as compared to free-cells with a concentration of 4.79 g/L and 0.37 (g/g) yield of lactic acid. The titer, yield, and productivity of lactic acid achieved in this study were comparably high with those reported in previous research as depicted in Table 4. In contrast, the cell immobilized with CSAC C had the lowest concentration of by-products, as shown in Fig. 11B. It is worthy of note that the larger pore volumes of CSAC C also indicates a larger capacity to hold a substance of interest i.e. by-product molecules. Hence, more by-product molecules were adsorbed onto the surfaces of CSAC C, which would ease the subsequent downstream processing. The usage of CSAC C appeared to be cost-effective in reducing operational cost for the secondary treatment in the purification process of lactic acid recovery.

**Table 3:** Characteristics of the CSAC used for immobilization carrier.

CSAC	A	B	C	D
Micropore volume (cm^3^/g)	0.35	0.36	0.36	0.38
Density (g/mL)	1.0	1.0	1.0	1.0
Specific surface area (BET-m^2^/g)	656.24	669.1	672	720.1
Micropore area (m^2^/g)	570.4	530.7	573.3	611.8

**Table 4:** Different carbon sources with the titer, yield, and productivity of lactic acid fermentation using different biocatalysts.

Carbon sources	Biocatalyst	Titer (g/L)	Yield (g/g)	Productivity (g/L/h)	References
Wheat stalks	*Bacillus coagulans*, MA-13	32.05	0.92	2.55	^52^
Carob bean	*Bacillus coagulans*	48.7	0.84	2.30	^53^
Microalgael biomass	*Bacillus coagulans Azu-10*	102.2	1.0	3.18	^54^
Glucose	*A. succinogenes ΔpflA strain*	43.05	0.3	0.72	^36^
Ricotta cheese whey	*Lactobacillus casei DSM 20,011*	43	0.536	1.05	^55^
Corn straw hydrolysate	*B. coagulans GKN316*	45.39	0.83	0.4725	^56^
PKC hydrolysate	*A. succinogenes* 130Z	31.64	0.92	0.88	This work

## Materials and methods

### Feedstock preparation

The PKC samples used in this study were freshly obtained from Kernel Crushing Plant Carey Island with permission from Sime Darby Plantation Berhad, Selangor, Malaysia. The PKC was dried in an oven at 60 °C for 18 hs to reduce the moisture content to ~ 10%^57^. The dried PKC samples were kept in a vacuum desiccator over silica gel until further use, following NREL (National Renewable Energy Laboratories) protocol for preparing samples for compositional analysis (NREL/TP-510-42,620)^58^.

### The compositional analysis of PKC

Table 5 shows the chemical compositions of PKC determined using the National Renewable Energy Laboratory (NREL, Colorado) Laboratory Analytical Procedures: Preparation of Samples for Compositional Analysis (NREL/TP-510-42,620) and Determination of Structural Carbohydrates and Lignin in Biomass (NREL/TP-510-42,618)^59^. As seen in Table 5, the raw PKC sample used in this study consisted of 10 wt% cellulose, 36.1 wt% hemicellulose and 20.6 wt% lignin. Both ethanol-soluble and water-soluble components were extracted from the PKC for 24 hs using the Soxhlet extractor (reflux rate: 4–5 syphon cycles per h). The PKC's extractive-free parts underwent an acid hydrolysis using an autoclave. The collected hydrolysate was vacuum filtered to separate the filtrate (soluble sugar monomers) from the Klason lignin.

**Table 5:** Chemical composition of the raw PKC.

Components	Dry weight (%)
Glucan	10.0 ± 0.3
Mannan	33.1 ± 0.4
Total carbohydrate	46.1 ± 1.0
Total extractives	21.5 ± 0.1
Lignin	20.6 ± 0.9
Ash	4.0 ± 0.1
Protein	11.8 ± 0.2
Moisture	5.42 ± 0.1

### Morphological structure of PKC

A field emission scanning electron microscope (FESEM Zeiss, Germany) was used to investigate the morphologies of both untreated and acid-pretreated PKC at its optimal level. Both samples were oven-dried before being mounted on aluminium stubs. In order to prevent sample surface charge, palladium was subsequently sputtered onto the samples using plasma.

### X-ray diffraction of PKC

The comparisons in the structures of the PKC before and after hydrolysis were determined by crystallinity index of the materials examined using X-ray diffraction (XRD). The samples were scanned using X-ray diffractometer (Bruker AXS D8 Advance, USA) with Ni-filter CuK radiation (= 1.541 ) at 40 kV and 40 mA at diffraction angle of 2θ = 0° to 60° with 0.25° per second speed. The crystallinity index (CrI) of sample was determined from the crystalline diffraction intensities and amorphous fraction using the Eq. (1):

1 $${\text{Crystallinity}}\;{\text{index}}\left( {{\text{CrI}}} \right) = \frac{{I_{002} - I_{{{\text{am}}}} }}{{I_{002} }} \times 100$$

where I_002_ is the intensity of crystalline fraction of _002_ plane at 2θ = 22.5° and I_am_ is the intensity of amorphous fraction at 2θ = 18.0°.

### Oxalic acid pretreatment of PKC

The pulverized PKC at 10% (w/v) (10 g PKC) was immersed in 100 mL of oxalic acid with different range of concentrations. In this work, the pretreatment conditions of PKC were manipulated i.e. reaction times (1-5 h) and oxalic acid concentrations (1–5%, w/v) with temperature fixed at 121 °C using autoclave. These parameter ranges were specified based on the results of a previous work on accomplishing hydrolysis under mild conditions^28^. Mannose recovery after acid pretreatment was calculated as follows (Eq. 2):

2 $$Mannose\;recovery\left( \% \right) = \frac{{{\text{Mannose}}\;{\text{recovered}}\;{\text{from}}\;{\text{hydrolysate}} \times 0.9}}{{{\text{Hemicellulose }}\;{\text{content}}\;{\text{in}}\;{\text{raw}}\;{\text{biomass}}}} \times 100\%$$

where 0.9 is the conversion factor of mannose to equivalent hemicellulose^30^.

### Enzymatic hydrolysis of pretreated PKC

The resulting pretreated PKC slurry was hydrolysed using a commercial cellulase (Cellic CTec2, Novozyme, Denmark) at 30 filter paper units (FPU) per gram of solid PKC loading. The pH of the pretreated slurry was first adjusted to 4.8 ± 0.2 using 10 M NaOH before enzyme was added. The mixture was incubated at 50 °C and agitated at 150 rpm for 72 h. At the end of incubation period, the liquid fraction (i.e. enzymatically-digested hydrolysate) was collected and filtered through a gauze cloth to remove the solid phase before being used in sugar analysis and subsequent fermentation. At this stage, the enzymatic digestibility was calculated as follows (Eq. 3):

3 $$Enzyme\;digestibility\left( \% \right) = \frac{{\left( {Glucose\;in\;enzymatic\;hydrolysate - glucose\;in\;acid\;pretreatment} \right) \times 0.9}}{Cellulose\;content\;in\;raw\;biomass} \times 100\%$$

where 0.9 is the conversion factor of glucose to equivalent cellulose.

### Batch fermentation with free cells using PKC hydrolysate

The hydrolysate from previous section was supplemented with 2.0 g MgCl_2_.6H_2_O, 1.5 g CaCl_2_.6H_2_O, 1.0 g NaCl, 3.3 g KH_2_PO_4_, 4.4 g K_2_HPO_4,_ 2.0 g urea, and 30.0 g yeast extract per litre of medium, respectively. The medium was adjusted to initial pH of 6.8 ± 0.2 using 5 M NaOH. A continuous pH control strategy was further employed using magnesium carbonate (MgCO_3_) supplementation method with a concentration of up to 30 g/L. Fermentation was performed in a 250-mL conical flasks by adding 10% (v/v) inoculum of *A. succinogenes* 130Z into a working volume of 100 mL. The culture was incubated at 37 °C and agitated at 200 rpm for the first 14 h and agitated at 40 rpm for subsequent 46 h^4^. The fermentation conditions were manipulated i.e. phases of cultivation (mono-phase and dual-phase), carbonates loading (10–50 g/L of MgCO_3_ and Na_2_CO_3_), and types of individual monosaccharides as substrate (glucose and mannose). All experiments were performed in triplicates.

### Characterisation of support material

A Physisorption Analyzer (Micromeritics ASAP 2010, Norcross, GA, USA) was used to characterise the carrier material used in this study. By using the N_2_ adsorption/desorption isotherm at 77 K, the Brunauer–Emmett–Teller (BET) surface area and pore properties of CSAC were determined.

A field emission scanning electron microscope (FESEM Zeiss, Germany) was used to investigate the morphologies of raw CSAC and immobilized cells on CSAC. Both samples were oven-dried before being mounted on aluminium stubs. In order to prevent sample surface charge, palladium was subsequently sputtered onto the samples using plasma.

### Repeated batch fermentation with immobilized cells using PKC hydrolysate

Coconut shell-activated carbon (CSAC) was used as an immobilizing agent. More durable CSAC carrier were prepared by mixing the CSAC with 24 h incubated growth medium culture containing PKC hydrolysate by 10% (v/v). Repeated batch fermentations for five consecutive runs were performed in 180 h using the same fermentation medium composition as in the case of free cells medium. The fermentation flasks were incubated at 37 °C and agitated at 200 rpm for the first 14 h and agitated at 40 rpm for subsequent 46 h. The CSAC-immobilized cells were washed with sterile NaCl (0.9 wt %) four times after each cycle of fermentation to remove non-viable cells. The cells were reused and supplemented with a new fresh medium for subsequent run of fermentations. The samples of fermentation were taken regularly until all the sugars have been used up^50^.

### Analytical methods

The sugars content recovered and organic acid formed were quantified using high performance liquid chromatography (HPLC) (Waters 2707, MA, USA) equipped with Phenomenex Rezex™ ROA column (300 mm × 7.8 mm) (Sunnyvale, USA). The column was maintained at 60 °C and the sugars and organic acids were eluted with 2.5 mM H_2_SO_4_ at a flow rate of 0.6 mL/min with the sample injection volume of 10 mL. The calibration curves were prepared by injecting the three concentrations (5, 10, and 15 g/L) of calibration standard solutions into the HPLC system at 10 µL. The apparatus was integrated with a Waters 2702 auto sampler, Waters 2414 refractive index detector (set at 40 °C) and Waters 1515 isocratic HPLC pump (Waters, MA, USA).

## Conclusion

Oxalic acid pretreatment on hemicellulose of PKC prior to saccharification has improved the enzymatic digestibility of PKC by 65.6% and yielding 34.32 g/L sugars. The optimal condition for fermentability of PKC hydrolysate was in dual phase cultivation of free cells supplemented with 30 g/L of MgCO_3_. Immobilized cells with coconut shell-activated carbon (CSAC) from different sizes in a dual phase, repeated batch cultivation in PKC hydrolysate has demonstrated to improve lactic acid concentration and productivity of up to 31.64 g/L and 0.88 g/L/h, respectively. The findings have demonstrated that the production of lactic acid from bioresources via mild oxalic acid pretreatment, enzymatic saccharification, and CSAC-immobilized cell fermentation provides an effective, sustainable, and flexible substitute for its petroleum-based competitors.

## Data Availability

All data generated or analysed during this study are included in this published article.
